# Change of Direction Speed Tests in Basketball Players: A Brief Review of Test Varieties and Recent Trends

**DOI:** 10.3389/fspor.2021.645350

**Published:** 2021-04-29

**Authors:** Takashi Sugiyama, Sumiaki Maeo, Toshiyuki Kurihara, Hiroaki Kanehisa, Tadao Isaka

**Affiliations:** ^1^Faculty of Sport and Health Science, Ritsumeikan University, Shiga, Japan; ^2^Research Organization of Science and Technology, Ritsumeikan University, Shiga, Japan

**Keywords:** agility, defensive, 180°-turn, cutting, reactive

## Abstract

Change of direction speed (CODS) is essential for basketball performance, extensively assessed by various tests. This review aimed to summarize the CODS test varieties for basketball players on publications until 2019 and identify recent trends regarding what types of tests have gained attention in the 2010s. Electronic literature searches were conducted using three databases with relevant keywords. 104 studies were found eligible, conducting CODS tests 159 times in total with 48 test varieties. To facilitate distinctions between the tests, each test was categorized into one of three types based on the distinctive movement characteristics and changing angles as follows: Defensive (involving lateral shuffling), 180°-turn (exerting only 180°-turns), and Cutting (performing diagonal- or side-cut). We then counted the number of publications and adopted times reported per year for each test, and calculated the adoption rate for each categorized test type. The first CODS test performed in basketball players was the *T*-Test, reported in 1991, and this was the most commonly adopted test (44/159 times). The 2010s saw abrupt increases in the number of publications (1990s-2000s-2010s: 5-9-90) and test varieties (4-7-44). The adoption rates in the 2010s were similar among the three types (i.e., Defensive/180°-turn/Cutting: 37%/30%/33%), with the Cutting type gradually increasing over the last three decades (1990s-2000s-2010s: 0%-9%-33%). These results suggest that while CODS performances in basketball players are increasingly studied with various tests, recent studies give equal weight to all of the three categorized test types, with increasing adoption of the Cutting type, to assess specific CODS performances.

## Introduction

Change of direction speed (CODS) is a determinant of athletic performance in various sport events (Sheppard and Young, 2006; Spiteri et al., 2014). This is also true for basketball, in which the players are repeatedly required to perform rapid accelerations and decelerations with sudden changes in directions in the small playing area (Scanlan et al., 2014a) compared to outfield sports such as soccer. Indeed, elite male and female basketball players have been shown to change movement types every 1-3 seconds during a game (Abdelkrim et al., 2007; Conte et al., 2015; Scanlan et al., 2015b). Thus, high CODS performance is considered a particularly critical physical demand in basketball players (Spiteri et al., 2015a; Stojanović et al., 2019a).

**Figure 1:**
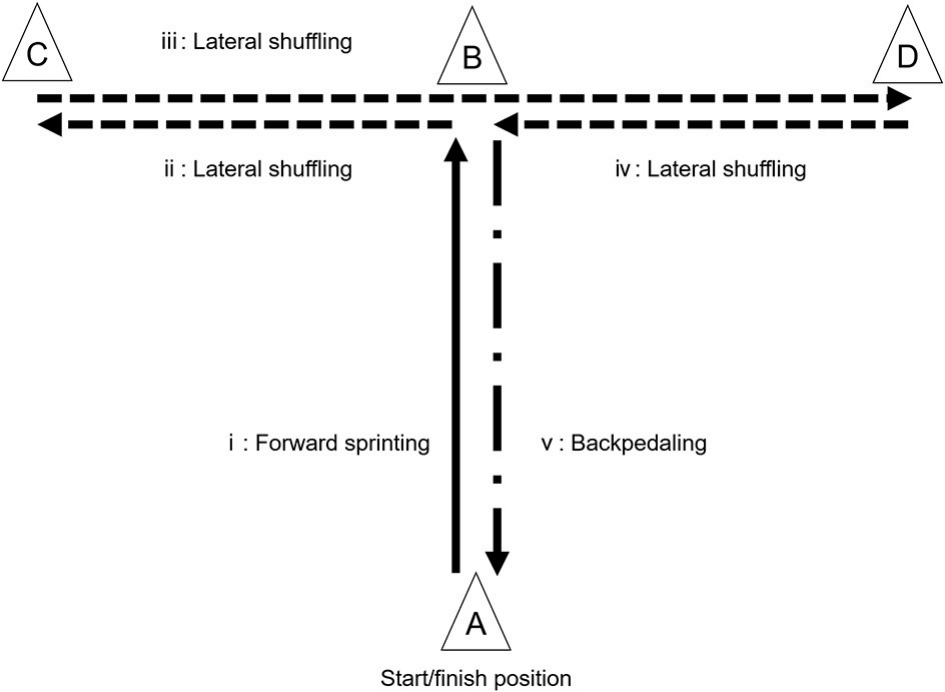
The procedure of the *T*-Test. The subjects perform i) forward sprinting from **(A)** to **(B)**, ii) lateral shuffling to the left from **(B)** to **(C)**, iii) lateral shuffling to the right from **(C)** to **(D)**, iv) lateral shuffling to the left from **(D)** to **(B)**, and v) backpedaling to the finish-position from **(B)** to **(A)**. The CODS performance is assessed by time to complete the task (i-v).

**Figure 2:**
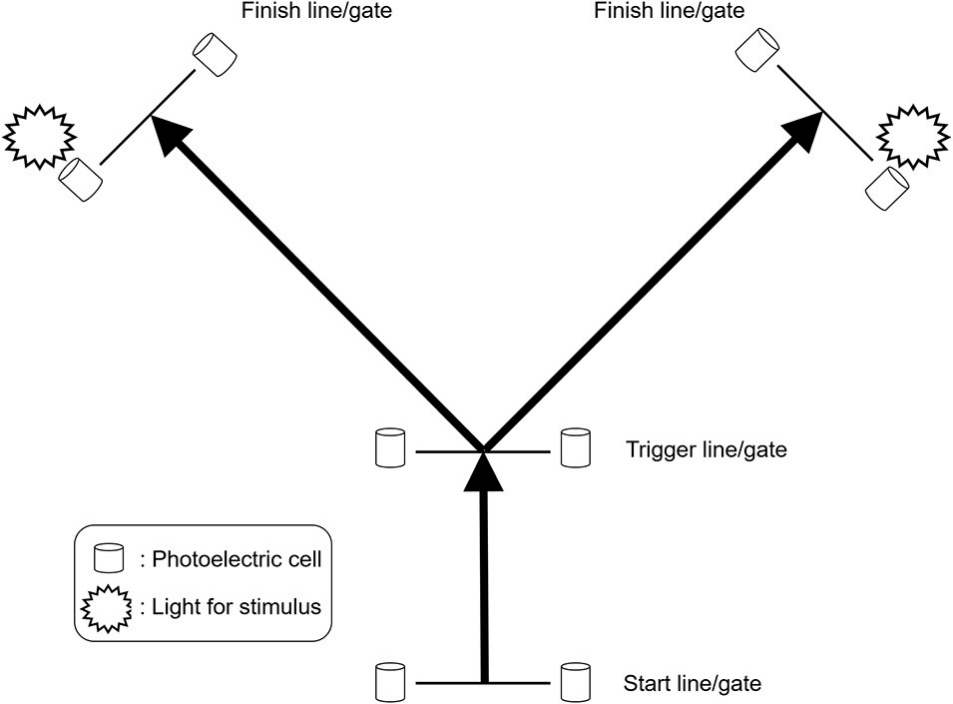
The typical protocol of a reactive test. This test requires decision-making in response to external stimuli before subjects change their direction. They run forward from the start line/gate to the trigger line/gate, at which point the light at the finish line/gate on either the right or left illuminates. The subjects must cut and sprint to the right or left finish line/gate depending on which light is illuminated. The performance is evaluated by the whole time (start-finish) and the time after the stimuli (trigger-finish).

The CODS performance in basketball players has been evaluated by various tests. One of the most typical CODS tests adopted in basketball is the *T*-Test (Figure 1). This test is designed to evaluate the performance of multiple movements, specifically characterized as involving defensive maneuvers (i.e., lateral shuffling and backpedaling) similar to basic basketball movements (Jakovljević et al., 2012; Stojanović et al., 2019a). Another CODS test in basketball is the Suicide-run (also known as Line-drill), which consists of four consecutive shuttle sprints with turns in 180° on a basketball court (running almost 140 m in total) (Carvalho et al., 2017; Doma et al., 2018), simulating a game-related motion at the transition between offensive and defensive actions. In these tests, examinees sprint a pre-determined course without reacting to external stimuli. On the other hand, reactive tests (Figure 2) requiring decision-making regarding the subsequent movement direction have been gaining attention since the early 2000s because such tests are thought to assess cognitive function, a determinant of performance in invasion sports (Young et al., 2002, 2015; Sheppard and Young, 2006) including basketball (Scanlan et al., 2014b). Moreover, studies in the 2010s suggested that strength and conditioning coaches should consider sport-specific “stop-and-go” scenarios, which are more frequent in small courts sports, when selecting CODS tests (Serpell et al., 2010; Sekulic et al., 2014). Taking these aspects into account, various types of CODS tests may well have been developed and implemented. However, it is unclear how many and what types/varieties of tests have been used to evaluate CODS performance in basketball players.

Several studies have already reviewed physical requirements in basketball including CODS performance, e.g., discussing the use of the *T*-Test and 505 (Ziv and Lidor, 2009; Wen et al., 2018; Mancha-Triguero et al., 2019), but only limited test varieties have been covered despite various other evaluation tests used. There also has been no review article solely focusing on CODS tests applied for basketball players. The rule changes of basketball in 2000 are suggested to have made competitive games faster and activity intensities during a match higher than before (Abdelkrim et al., 2007). This tempted us to assume that the development of new CODS tests might have been prompted to more precisely examine the CODS performances of basketball following the rule changes. Thus, the current brief review aimed to summarize the varieties of the CODS tests adopted for basketball players on publications until 2019, and identify recent trends regarding what types of tests have gained attention in the 2010s. To this end, we first identified all individual CODS tests and then classified them into three types based on their distinctive movements often seen in basketball games. A comprehensive examination of the CODS tests adopted in basketball would provide useful information for basketball players and their strength and conditioning coaches to select tests fit for the purpose to evaluate the CODS performance of the players.

## Materials and Methods

### Literature Search Strategy

Electronic database searches were performed using PubMed, Web of Science, and SPORTDiscus in the title/abstract with keywords relating to CODS tests for basketball players. The search in the Web of Science was also conducted in keywords and Sports Sciences field. The last search was performed in January 2020 on publications until the end of 2019. A search formula included the following terms: (“change of direction” OR “changes of direction” OR agility OR “cutting maneuver” OR “cuts maneuver” OR turn OR turns OR turning OR step OR steps OR stepping OR start OR stop OR stops OR stopping OR swerve OR footwork OR braking OR “breaking ability” OR “cross step” OR “cross stepping” OR “lateral cutting” OR “lateral cut” OR “side step” OR “sidestep” OR “side stepping” OR avoid^*^ OR “avoidance movement” OR “avoidance strategy” OR “repeated sprint ability” OR “change of pace” OR fake OR juke OR “juke action” OR curve OR shuffling) AND (test OR dill) AND (basketball).

### Screening and Eligibility Criteria

The selection of studies was carried out through three consecutive screening phases. In the first phase, duplicated literature was removed. In the second phase, relevant articles based on the title/abstract were assessed with inclusion criteria. Finally, exclusion criteria were used to assess the eligibility of full-text articles. This review incorporated articles if they fulfilled all of the following inclusion criteria: (a) written in English, (b) subjects included basketball players, and (c) implemented a CODS/agility test. This study excluded the articles accepted in the second phase if they did not meet any of the following criteria: (a) research with full-text published in English, (b) implementation of the test involved at least one change of direction without dribbling (as it assesses dribble skills), (c) the test was completed in less than 40 s and 140 m (i.e., the Suicide run was included but the Yo-Yo test was excluded) and evaluated by the sprint time, speed, or the number of repeated times, (d) the CODS test was conducted without a rest or jog during the test (i.e., intermittent types were excluded), (e) the procedure and content of the test included clear explanation (in figure and/or text or with a reference accessible via internet), (f) subjects were ≥12 and ≤65 years old to be considered as basketball players (i.e., studies on only elementary school students or older adults who participated in the masters games were excluded), (g) information about the results was sufficiently clear, and (h) data for basketball players could be identified/extracted from other sports players.

### Categorization and Chronological Classification of the Tests

Firstly, the contents and individual names of the selected CODS tests were identified. For some tests, researchers have used different names when referring to the same content (e.g., *T*-Test vs. *T*-test agility vs. agility *T*-test). In such cases, we used the most common name (e.g., *T*-Test). In contrast, some tests have been named the same even though the contents such as running distance were slightly different. In these cases, we re-named each test based on its content (e.g., 5 m^*^2-shuttle vs. 7.5 m^*^2-shuttle, which had been both named 180°-COD in previous studies). To facilitate a distinction between test types, we categorized individual CODS tests into one of three types as follows exclusively in this order; (a) Defensive: involving lateral movement with/without backpedaling, (b) 180°-turn: exerting only 180°- turns, and (c) Cutting: performing diagonal- or side-cut with/without 180°- turns. These distinctive movements (Jakovljević et al., 2012; Stojanović et al., 2019a) and angles of turns/cuts (Carvalho et al., 2011; McCormick et al., 2014; Gonzalo-Skok et al., 2015; Spiteri et al., 2015a) are considered the basic motions in basketball.

**Figure 3:**
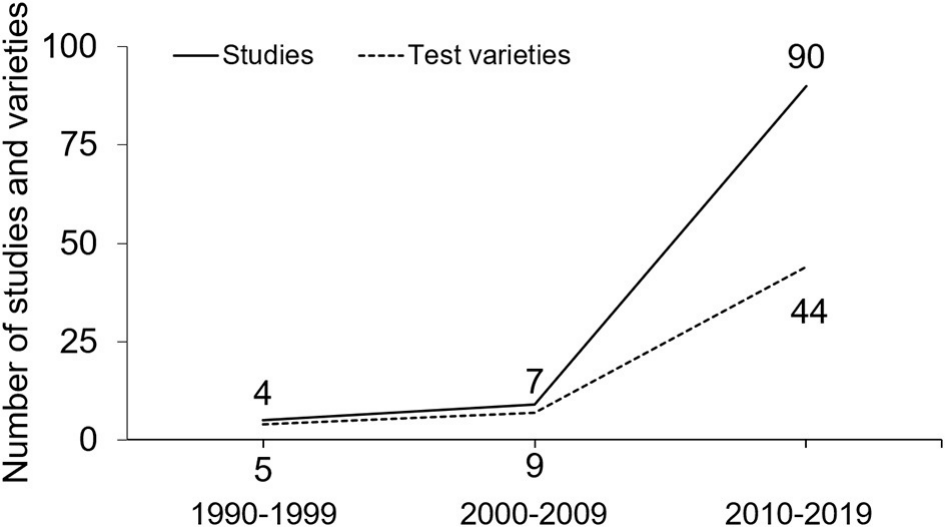
The numbers of studies and test varieties assessing CODS in basketball players shown at ten-year intervals.

**Figure 4:**
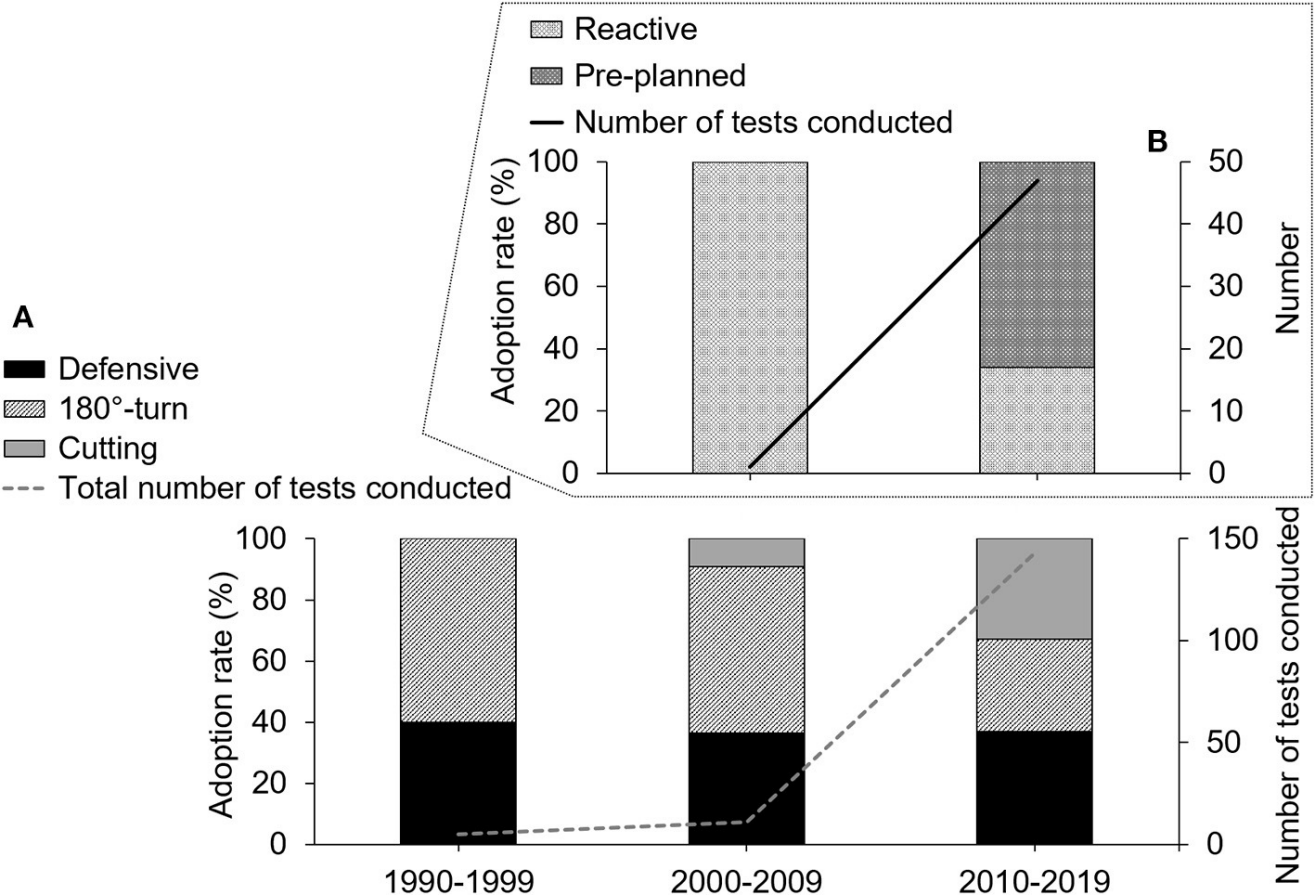
The adoption rate of each test type (bar graph/left-axis) and the total number of tests conducted (line graph/right-axis) for the three categorized types (defensive vs. 180°-turn vs. cutting: **(A)** and the two subcategorized types (pre-planned vs. reactive: **(B)** shown at ten-year-intervals. Note that reactive types were only used in the cutting type in 2000s and 2010s (detailed in Table 1).

Additionally, in each of the three categorized test types, individual tests were further subcategorized into either a pre-planned type (i.e., performing a change of direction without reacting to external stimuli) or a reactive type (i.e., requiring decision-making regarding the subsequent movement direction). The number of studies, individual test varieties, their categorized types, and subcategorized types were counted on a yearly basis, and are summarized at 5-year intervals in Table 1 (for detailed information) and shown at 10-year intervals in Figures 3, 4 (for ease of visual interpretation). A test was counted only once within the same study no matter how many times it was conducted (e.g., for reliability measurement purposes). Finally, the adoption rate (%) of each test type was calculated in the three categorized types (i.e., defensive vs. 180°-turn vs. cutting), as well as in the two subcategorized types (i.e., pre-planned vs. reactive), by dividing the number of each test type by the total number.

**Table 1:** A list of CODS tests in basketball players and their numbers adopted from 1990 to 2019 shown at every five-year interval.

	**Categories**		**Years**						**Whole**
**No**.		**Sub-categories / Individual tests [Ref.]**	**1990–1994**	**1995–1999**	**2000–2004**	**2005–2009**	**2010–2014**	**2015–2019**	
	**Defensive**								
		*Pre-planned*	*(1)*	*(1)*	*(0)*	*(4)*	*(19)*	*(34)*	***(59)***
1		T-Test (Hoffman et al., 1991, 1999; Delextrat and Cohen, 2008, 2009; Chaouachi et al., 2009; Abdelkrim et al., 2010; Köklü et al., 2010, 2011; Jakovljević et al., 2011a,b, 2012; Alemdaroglu, 2012; Arazi et al., 2012; Asadi and Arazi, 2012; Asadi, 2013, 2016; Lehnert et al., 2013; Sekulic et al., 2013, 2017; Delextrat and Martinez, 2014; Spiteri et al., 2014, 2015a; Atanasković et al., 2015; Zarić, 2014; Fort-Vanmeerhaeghe et al., 2016; Freitas et al., 2016, 2019; SiSic et al., 2016; Soslu et al., 2016; Asadi et al., 2017; Mtsweni et al., 2017; Myles et al., 2017; Buscà et al., 2018; Garcia-Gil et al., 2018; Luis et al., 2018; Maggioni et al., 2018; Arede et al., 2019a; Guimarães et al., 2019a,b; Meszler and Váczi, 2019; Mitić et al., 2019; Ramos et al., 2019a,b; Scanlan et al., 2019)	1	1		3	15	24	***44***
2		Lane (Kucsa and Mačura, 2015; Izzo and Varde'i, 2018; Cui et al., 2019; Stojanović et al., 2019a,b; Townsend et al., 2019)						6	***6***
3		SEMO (Marzilli, 2008)				1			***1***
4		Run-shuffle-run (Stojanović et al., 2019a)						1	***1***
5		Lane-arrow-closeout (Stojanović et al., 2019a)						1	***1***
6		Planned-agility (Delextrat et al., 2015)						1	***1***
7		Lateral-shuffle-test (Delextrat and Martinez, 2014; McCormick, 2014; McCormick et al., 2016)					2	1	***3***
8		Zigzag-side-shuffling (Erčulj et al., 2011)					1		***1***
9		Defensive-lateral-shuffle (McCormick et al., 2014)					1		***1***
		*Reactive*	*(0)*	*(0)*	*(0)*	*(0)*	*(0)*	*(0)*	***(0)***
		Not Available							
	**180°-turn**								
		*Pre-planned*	*(0)*	*(3)*	*(3)*	*(3)*	*(15)*	*(28)*	***(52)***
10		Suicide-run (Hoffman et al., 1999; Hoare, 2000; Delextrat and Cohen, 2009; Carvalho et al., 2011, 2017, 2019; Fort-Vanmeerhaeghe et al., 2016; Mtsweni et al., 2017; Myles et al., 2017; Doma et al., 2018; Maggioni et al., 2018; Ramos et al., 2019a; Stojanović et al., 2019b)		1	1	1	1	9	***13***
11		505 (Van Gelder and Bartz, 2011; Cvorović, 2012; Spiteri et al., 2014, 2015a, 2019; Dos'Santos et al., 2018; Stojanović et al., 2019a)					3	4	***7***
12		Pro-agility / 20 yard test (Locke et al., 1997; Sekulic et al., 2013; Arede et al., 2019b; Aschendorf et al., 2019; Banda et al., 2019; Stojanović et al., 2019a; Townsend et al., 2019)		1			1	5	***7***
13		5 m*2-shuttle (Fort-Vanmeerhaeghe et al., 2015; Gonzalo-Skok et al., 2019)						2	***2***
14		5.8 m*2-shuttle (Cook et al., 2004)			1				***1***
15		7.5 m*2-shuttle (Gonzalo-Skok et al., 2017)						1	***1***
16		22.86 m-shuttle (Greene et al., 1998)		1					***1***
17		5.8 m*3-shuttle (Hoare, 2000)			1				***1***
18		5 m*4-shuttle (Peña et al., 2018)						1	***1***
19		9 m*4-shuttle (Asadi and Arazi, 2012, 2018; Asadi, 2013)					2	1	***3***
20		10 m*4-shuttle (Huang et al., 2018)						1	***1***
21		15 m*4-shuttle (Jakovljević et al., 2011a, 2012)					2		***2***
22		10 m*5-shuttle (Boone and Bourgois, 2013)					1		***1***
23		Suicide-run(half) (Jakovljević et al., 2017)						1	***1***
24		5 m*6-shuttle (Erčulj and Bračič, 2009; Erčulj et al., 2009, 2010, 2011; Štrumbelj and Erčulj, 2014)				2	3		***5***
25		5 m*10-shuttle (Kucsa and Mačura, 2015; Pion et al., 2015)						2	***2***
26		Agility with 180-degree turn (Sekulic et al., 2013; SiSic et al., 2016)					1	1	***2***
27		Forward-backward running agility (Sekulic et al., 2013)					1		***1***
		*Reactive*	*(0)*	*(0)*	*(0)*	*(0)*	*(0)*	*(0)*	***(0)***
		Not Available							
	**Cutting**								
		*Pre-planned*	*(0)*	*(0)*	*(0)*	*(0)*	*(9)*	*(22)*	***(31)***
28		Illinois (Asadi and Arazi, 2012; Asadi, 2013, 2016; Šimonek et al., 2016; Asadi et al., 2017; Bouteraa et al., 2018; Meszler and Váczi, 2019)					2	5	***7***
29		V-cut (Gonzalo-Skok et al., 2015, 2017, 2019)						3	***3***
30		COD-sprint (Scanlan et al., 2018; Ramirez-Campillo et al., 2019)						2	***2***
31		Zigzag-agility-drill (Jakovljević et al., 2011a, 2012; SiSic et al., 2016)					2	1	***3***
32		Zig-zag test (Sekulic et al., 2013)					1		***1***
33		45deg-sidestep-cutting (Lam et al., 2017)						1	***1***
34		L-run / 3-cones (Atanasković et al., 2015)						1	***1***
35		Compass (Stojanović et al., 2019a)						1	***1***
36		T-shaped–Forward Sprint (Miloski et al., 2015)						1	***1***
37		COD-test (Doma et al., 2018)						1	***1***
38		Cross-over sprint (Jakovljević et al., 2017)						1	***1***
39		Control movement test (Jakovljević et al., 2017)						1	***1***
40		Closed-skill-agility (Scanlan et al., 2014a,b, 2015a)					2	1	***3***
41		Y-shaped-agility (Lockie et al., 2014a,b; Jeffriess et al., 2015)					2	1	***3***
42		Pre-planned basketball-specific (Sekulic et al., 2017; Pehar et al., 2018)						2	***2***
		*Reactive*	*(0)*	*(0)*	*(0)*	*(1)*	*(5)*	*(11)*	***(17)***
43		Open-skill-agility (Gabbett et al., 2008; Scanlan et al., 2014a,b, 2015a, 2016)				1	2	3	***6***
44		Y-shaped-agility (Lockie et al., 2014a,b; Jeffriess et al., 2015; Horička and Šimonek, 2019)					2	2	***4***
45		Nonplanned basketball-specific (Sekulic et al., 2017; Pehar et al., 2018)						2	***2***
46		Offensive-agility (Spiteri et al., 2019)						1	***1***
47		Defensive-agility (Spiteri et al., 2019)						1	***1***
48		Multidirectional-agility (Spiteri et al., 2014, 2015a,b)					1	2	***3***
		Total number of CODS tests conducted	1	4	3	8	48	95	**159**
		Number of test varieties	1	4	3	5	21	37	-
		Defensive	1	1		4	19	34	
		180°-turn		3	3	3	15	28	
		Cutting				1	14	33	

*CODS: change of direction speed; Not available indicates there were no reactive tests in the Defensive and 180°-turn types*.

## Results

### Number of Studies and Test Varieties

A total of 798 studies was initially retrieved, and then finally, 104 pieces of literature conducting at least one CODS test were selected in the light of inclusion/exclusion criteria. The CODS tests were conducted 159 times in total, with 48 test varieties in the last three decades (Table 1). From 1990 to 1999, the number of studies examining CODS was five, with every study adopting only one test (Figure 3). The number of studies adopting more than one test has increased since 2000s, and some studies have adopted three or more tests since 2010s.

The first CODS evaluation in basketball players was reported in 1991 and conducted by the *T*-Test, belonging to the Defensive type, which also was the most commonly adopted individual test until 2019 (44/159 times, Table 1). From 1990 to 2004 (early 15 years), only the Defensive and 180°-turn types were adopted and then Cutting type emerged in the late 2000s. The numbers of studies and test varieties explosively increased in the 2010s (Figure 3). The reactive type has been used since the late 2000s, but exclusively in the Cutting type (Table 1).

### Adoption Rate of Each Test Type

Based on the three categorized types, the 180°-turn type (60%) was often used in the 1990s compared to the Defensive type (40%) with no adoption of the Cutting type (0%) (Figure 4A). In the 2000s, the Cutting type was used but only in one study (9%), not affecting the dominance of the 180°-turn type (55%) followed by the Defensive type (36%). The most recent adoption rate in the 2010s noticeably changed from the previous two decades, with the Cutting type (33%) comparable to the 180°-turn (30%) and Defensive types (37%), each accounting for ~one-third of their sum (Figure 4A). From the viewpoint of the subcategories, the adoption rate of the reactive vs. pre-planned type within the Cutting type in the 2000s was 100 vs. 0% (but the former was used only once), and that in the 2010s was 34 vs. 66% (16 vs. 31 times) (Figure 4B).

## Discussion

The main findings obtained here were that (1) the CODS tests were conducted with 48 test varieties from 1991 to 2019, with abrupt increases in their varieties after 2010, (2) in the last decade, each of the Defensive, 180°-turn, and Cutting types had similar adoption rates (each about one-third of their sum), and (3) the *T*-Test was the first and most commonly conducted individual CODS test.

**Figure 5:**
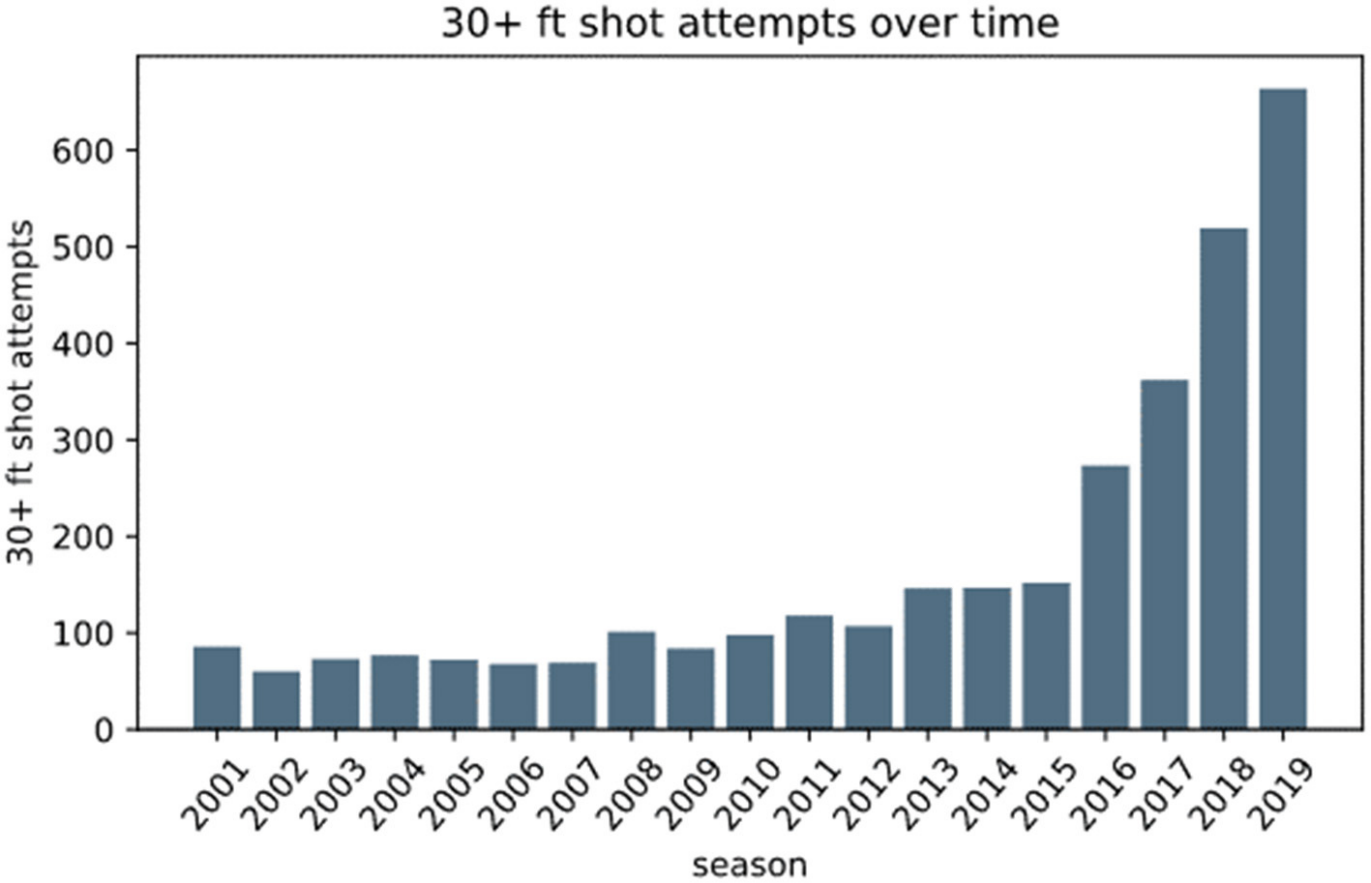
Shift in the number of attempted three-point shots from > 30 feet away from the basket goal in NBA (Cheema, 2019), reprinted with the author's permission.

The number of CODS test varieties in basketball players abruptly increased after 2010 (Figure 3). In fact, only four and seven test varieties were adopted in the 1990s and 2000s, respectively, while it creased to 44 in the 2010s (Figure 3). The increased test varieties may be at least partly attributed to the changes in the rules and game tactics of basketball. In 2000, International Basketball Federation shortened the time for the shot clock violation from 30 to 24 s and the time for the backcourt violation from 10 to 8 s (BASKETREF.COM: Rules History 2000-2010, Rules, n.d.). These changes are suggested to have made the games faster and necessitated the players to change direction more often than before (Abdelkrim et al., 2007). From a tactical point of view, the number of attempted three-point shots per game, particularly from > 30 feet away from the goal (Figure 5) (Cheema, 2019), has been increasing year by year in the National Basketball Association League (NBA) (Shea, n.d.). Consequently, defensive players are required to move more widely and quickly to interfere with the movement of the offensive players, which often results in giving up space for the other opponents to cut into. Such changes in the expanded playing area for both offensive and defensive players, as well as increased playing intensity during a game, might have been a factor yielding the diversity in the tests. To support this, CODS tests involving cutting maneuvers to various directions, belonging to the Cutting type, have been used only recently (since the late 2000s, Table 1). Moreover, new CODS tests may have been developed by researchers with the help of accumulating sports science knowledge, as can be seen from the increasing number of publications and varieties of CODS tests in basketball (Figure 3) and likely in other sports as well (Serpell et al., 2010), in an effort to specifically evaluate the multi-faced CODS performances. Thus, the CODS test varieties may have been increased with the progress of sports science knowledge in order for coaches/researchers to better assess players' physical demands, which have been updated with changes in basketball rules and tactics.

The present study classified the 48 test varieties into three types based on the basketball-related movements to summarize the test types and recent trends. While there has been a sharp increase in the number of the conducted CODS tests and varieties in the last decade, the adoption rate was similar among the three test types, each composing about one-third of the sum (Figure 4). This may be because the content of each test type reflects a distinctive change of direction movement required in basketball. More specifically, the Defensive type involves lateral shuffling, which is one of the basic defensive movements in basketball (McCormick et al., 2014). The 180°-turn type well represents switching between offense and defense (Carvalho et al., 2011). The Cutting type replicates offensive movement patterns of cutting to diagonal or side directions (Gonzalo-Skok et al., 2015; Spiteri et al., 2015a), such as when eluding their opponent, which have been recently gaining attention as discussed earlier. It is worth noting that sprint speed together with eccentric leg muscle strength explained 67% of the inter-individual variance in the 505-test (180°-turn) performance (Jones et al., 2009). More interestingly, the combination of eccentric leg muscle strength and cognitive function, but not sprint speed, was selected as a strong predictor (70%) of the *T*-Test (Defensive) performance (Naylor and Greig, 2015). These may be because both tests involve “stop-and-go” scenarios, therefore requiring high eccentric leg muscle strength to decelerate, with the cognitive function rather than sprint speed likely playing some role in conducting complex defensive maneuvers in the *T*-Test. For the pre-planned Cutting type, kinematic and kinetic parameters during the task are reported to be associated with the V-shaped-cut test performance (Marshall et al., 2014), suggesting the importance of body control and skills. It is also worth mentioning that the cognitive function alone, with no additional contribution of other factors, explained 29% of the Reactive-Y-shaped test (reactive Cutting type, discussed later) performance (Naylor and Greig, 2015). Collectively, these findings suggest that different CODS tests (types) can evaluate different aspects of CODS performance. Importantly, there is currently no single test that fulfills all of the above-mentioned basketball-related movements (in either a reactive or pre-planned scenario) to assess various CODS performances. For strength and conditioning coaches, therefore, it is reasonable to select multiple tests from different perspectives (e.g., from each of the three types categorized in this study) based on the test contents and CODS performance of interest.

On an individual test basis, the *T*-Test was firstly and most frequently used in basketball from 1991 to 2019 (44/159 times, Table 1). This may be attributed to the following reasons. The *T*-Test involves several movements starting with forward sprinting (acceleration) and rapid deceleration, lateral shuffling, and then backpedaling, all of which are often seen in basketball (Jakovljević et al., 2012; Stojanović et al., 2019a). The reliability and validity of the *T*-Test have also been confirmed (Pauole et al., 2000). Furthermore, there is an advantage that the obtained data of this test can be compared to those of many previous studies on basketball players (see Table 1). Thus, the *T*-Test can be considered as the standard, albeit not fully comprehensive (as discussed above), CODS test in basketball.

Basketball players are often required to change direction in response to the opponents' movement direction (i.e., in a reactive manner) (Spiteri et al., 2014). It is reported that a reactive, but not pre-planned, CODS test revealed a better performance for semi-professional than amateur players (Lockie et al., 2014b) and regular than non-regular players (Scanlan et al., 2015a). This suggests that the performance assessed by reactive type tests depends more on cognitive function than physical factors (Naylor and Greig, 2015; Scanlan et al., 2015a). Such findings highlight the usefulness of reactive type tests in assessing CODS performance reflecting cognitive functions, which are essential for athletes to gain an advantage during a competitive game (Young et al., 2002, 2015; Sheppard and Young, 2006). Interestingly, the reactive type was found to be used in basketball from the late 2000s but only in the Cutting type so far (Table 1). Furthermore, its adoption rates in the 2010s was lower than the pre-planned type (34 vs. 66%, Figure 4B). The low prevalence of the reactive type (17/159 times in all tests: 11%) in basketball may be simply because this kind of test is relatively new, but also because it takes greater time/effort to set up a measurement system compared to the pre-planned type. Nevertheless, considering the potential benefit of the reactive type in assessing CODS performance with cognitive functions, future studies are expected to adopt/develop reactive type tests more often than before in not only the Cutting type but also Defensive and 180°-turn types.

## Limitations

This review has some limitations. First, we classified the CODS tests into three types based on the distinctive movements and the angles of the directional changes in CODS tests. However, CODS tests can be classified by other criteria, e.g., the duration or the number of directional changes during the test. Therefore, it should be pointed out that the categorization of this study is not the only way to distinguish one CODS test type from another. Categorizing CODS tests from various perspectives will provide useful information and needs further research. This study could be used as a foundation for such work. Second, we excluded the CODS tests that took more than 40 sec/140 meter in total to complete or had a rest/jog phase during the test, as such tests are usually used to evaluate endurance capacity rather than the CODS performance (Haff and Triplett, 2015). Similarly, tests that involved dribbling, likely reflecting the skill, were not included. On the other hand, we acknowledge that the endurance capacity as well as the dribbling skill are essential physical demands for basketball players to achieve high performance (Garcia-Gil et al., 2018; Ramirez-Campillo et al., 2019). It is worth noting that some studies have used CODS tests involving intermittent/endurance running (Scanlan et al., 2012; Staunton et al., 2017) or with dribbling during the CODS test (Erčulj et al., 2017; Scanlan et al., 2018; Ramirez-Campillo et al., 2019) in basketball players. Thus, further research considering these aspects is warranted to develop the optimum CODS test for basketball players, based on various categorizations. Finally, this review did not take into account the profiles of the basketball players, such as sex, age, and performance level, examined in each study. If these factors are considered, the results on the adoption rates of the three types of CODS tests would differ from those obtained in the current study. In the present circumstance, however, the number of publications is imbalanced between such sub-groups for discussing the data in a quantitative manner (e.g., male vs. female; 70:37, Amateur vs. Professional; 71:34). It is necessary to accumulate more evidence to elucidate whether the type of CODS tests should be selected in accordance with the profiles of the basketball players examined.

## Conclusion

In summary, while the CODS performance in basketball players are increasingly studied with various tests, recent studies appear to give equal weight to all of the three categorized test types of the Defensive, 180°-turn, and Cutting, to assess specific CODS performances. The reactive type tests have been used since the late 2000s in addition to the traditional pre-planned type tests, but their prevalence is still low and expected to increase in the future.

### Practical Application

The findings obtained here will be useful information for strength and conditioning coaches to select appropriate tests for evaluating the CODS performance of basketball players. There is good evidence that the CODS performance assessed by the Defensive type (e.g., the *T*-Test) is associated with muscle strength and cognitive function, while that of the 180°-turn type (e.g., 505-test) is mainly determined by sprint speed and muscle strength. Further, the performance of the pre-planned (e.g., V-shaped-cut) and reactive (e.g., Reactive-Y-shaped) Cutting types may well reflect body control/skills and cognitive function, respectively. However, currently no single CODS test contains every basketball-related movement characteristic in either a pre-planned or reactive scenario. Hence, in basketball, we propose that strength and conditioning coaches select multiple tests from different types based on the contents such as movement motions, cutting angles, and decision-making components to evaluate the specific CODS performance from several perspectives.

## Author Contributions

All authors contributed to the review conception and design. TS searched the literature and selected relevant articles. TS, SM, TK, HK, and TI performed data interpretation. TS wrote the first draft of the manuscript. All authors edited and revised on previous versions of the manuscript. All authors read and approved the final manuscript.

## Conflict of Interest

The authors declare that the research was conducted in the absence of any commercial or financial relationships that could be construed as a potential conflict of interest.
